# Pilot randomised clinical trial of an eHealth, self-management support intervention (iVERVE) for stroke: feasibility assessment in survivors 12–24 months post-event

**DOI:** 10.1186/s40814-020-00706-x

**Published:** 2020-11-07

**Authors:** Dominique A. Cadilhac, Nadine E. Andrew, Doreen Busingye, Jan Cameron, Amanda G. Thrift, Tara Purvis, Jonathan C. Li, Ian Kneebone, Vincent Thijs, Maree L. Hackett, Natasha A. Lannin, Monique F. Kilkenny, on behalf of the ReCAPS investigators

**Affiliations:** 1 Stroke and Ageing Research, Department of Medicine, School of Clinical Sciences at Monash Health, Monash University, Level 3 Hudson Institute Building, 27-31 Wright Street, Clayton, VIC 3168 Australia; 2 Florey Institute of Neuroscience and Mental Health, The University of Melbourne, Heidelberg, VIC Australia; 3 Department of Medicine, Peninsula Clinical School, Central Clinical School, Monash University, Frankston, VIC Australia; 4 NPS MedicineWise, Sydney, NSW Australia; 5 School of Nursing and Midwifery, Monash University, Clayton, VIC Australia; 6 Faculty of Engineering, Monash University, Clayton, VIC Australia; 7 Discipline of Clinical Psychology, Graduate School of Health, University of Technology Sydney, Ultimo, NSW Australia; 8 Department of Medicine, Austin Health, Heidelberg, VIC Australia; 9 The George Institute for Global Health, Faculty of Medicine, University of New South Wales, Sydney, NSW Australia; 10 Faculty of Health and Wellbeing, The University of Central Lancashire, Preston, UK; 11 Department of Neurosciences, Central Clinical School, Monash University, Melbourne, VIC Australia; 12 Department of Allied Health (Occupational Therapy), Alfred Health, Melbourne, VIC Australia

**Keywords:** Stroke, eHealth, Feasibility studies, Healthcare technology

## Abstract

**Background:**

Electronic communication is used in various populations to achieve health goals, but evidence in stroke is lacking. We pilot tested the feasibility and potential effectiveness of a novel personalised electronic self-management intervention to support person-centred goal attainment and secondary prevention after stroke.

**Methods:**

A phase I, prospective, randomised controlled pilot trial (1:1 allocation) with assessor blinding, intention-to-treat analysis, and a process evaluation. Community-based survivors of stroke were recruited from participants in the Australian Stroke Clinical Registry (AuSCR) who had indicated their willingness to be contacted for research studies. Inclusion criteria include 1–2 years following hospital admission for stroke and living within 50 km of Monash University (Melbourne). Person-centred goals were set with facilitation by a clinician using a standardised template. The intervention group received electronic support messages aligned to their goals over 4 weeks. The control group received only 2–3 electronic administrative messages. Primary outcomes were study retention, goal attainment (assessed using Goal Attainment Scaling method) and satisfaction. Secondary outcomes were self-management (Health Education Impact Questionnaire: 8 domains), quality of life, mood and acceptability.

**Results:**

Of 340 invitations sent from AuSCR, 73 responded, 68 were eligible and 57 (84%) completed the baseline assessment. At the goal-setting stage, 54/68 (79%) were randomised (median 16 months after stroke): 25 to intervention (median age 69 years; 40% female) and 29 to control (median age 68 years; 38% female). Forty-five (83%) participants completed the outcome follow-up assessment. At follow-up, goal attainment (mean GAS-T score ≥ 50) in the intervention group was achieved for goals related to function, participation and environment (control: environment only). Most intervention participants provided positive feedback and reported that the iVERVE messages were easy to understand (92%) and assisted them in achieving their goals (77%). We found preliminary evidence of non-significant improvements between the groups for most self-management domains (e.g. social integration and support: *β* coefficient 0.34; 95% CI − 0.14 to 0.83) and several quality-of-life domains in favour of the intervention group.

**Conclusion:**

These findings support the need for further randomised effectiveness trials of the iVERVE program to be tested in people with new stroke.

**Trial registration:**

ANZCTR, ACTRN12618001519246. Registered on 11 September 2018—retrospectively registered.

**Supplementary Information:**

The online version contains supplementary material available at 10.1186/s40814-020-00706-x.

## Key messages on feasibility

- What uncertainties about feasibility existed prior to this study?

A novel, co-designed electronic self-management intervention to support person-centred goal attainment and secondary prevention after stroke was developed that would enable tailoring and the ability to personalise support messages. Prior to testing for effectiveness in a randomised controlled trial (RCT), we sought to assess the feasibility of implementing the complex (i.e. has multiple components) intervention design, the electronic randomisation and data collection processes, and obtain feedback from survivors of stroke to inform the design of future RCTs.

- What are the key feasibility findings from this study?

Feasibility was determined from sufficient participants completing the trial (> 80%), feedback that the messages were useful and educational, requests to ‘stop’ messages were few and there was evidence of goal attainment and satisfaction with the program. System failures for sending messages were not detected. Practical barriers in this early test phase included the length of the intervention being too short (4 weeks) to fully understand all the implications of running the full pre-planned 12-week program and the inclusion of participants who had their stroke over 1 year before receiving the intervention.

- What are the implications of the feasibility findings on the design of the main study?

The results provided information for us to refine our study processes and procedures related to setting personalised goals and assignment of messages and an indication the intervention would be more relevant to survivors of stroke following discharge from the hospital after a new stroke. A mixed-method process evaluation will be performed alongside the future RCTs with a phase II study to enable testing of the new procedures in recruiting patients directly from hospitals rather than from the community and to assess responder burden and retention rates from a longer intervention period.

## Background

Stroke is a leading cause of global disease burden [1]. The presence of physical disability, loss of employment, inability to participate in pre-stroke activities, social isolation, anxiety [2] and depression [3] make returning to the community difficult [4]. This can be further complicated by the need to manage the risk factors that caused the stroke to avoid further vascular events. These ongoing physical, physiological and psychosocial impacts are associated with unplanned readmission, reduced participation and quality of life, and unmet needs across a range of domains [5, 6]. For people with a chronic condition such as stroke, adjustments such as learning new behaviours or modifying one’s lifestyle are necessary, but also challenging [7]. The ease with which adjustments occur is multifaceted and relies, in part, on a person’s self-efficacy, including beliefs and confidence about their capabilities in performing various everyday activities. Self-efficacy specifically influences health behaviours, the types of goals an individual will set and their ability to attain them [8]. Facilitating self-efficacy, for example by providing self-management support, may influence how much effort individuals invest in achieving their health goals and their resilience when faced with difficulties or failure [7]. Jones and Riazi have identified that self-efficacy is an important variable associated with outcomes such as quality of life, perceived health status, depression, activities of daily living and aspects of physical functioning [7]. One approach to enhancing self-efficacy in people living with stroke is through self-management programs [7].

To maximise the effectiveness of self-management programs, effort must be made to set well-defined recovery goals and ensure goals are person-centred (and not clinician imposed) [9]. Having ongoing support to achieve these goals is also key for goal attainment [10, 11], but often there are barriers faced once people return to community living after discharge from the hospital. The scale of providing such support to survivors of stroke living in the community requires innovation in approach and delivery. Research testing novel approaches for providing support programs is required, including new or improved technology-based products and processes. Building the evidence base for successful solutions may provide people with stroke who are living in the community with the necessary assistance to continue with their recovery and optimise their secondary prevention management [12].

The increase in the use of mobile phones and personal computers/tablets represents an important resource for lifestyle behaviour change and disease management [13, 14]. Online eHealth support tools, accessible from portable devices or personal computers, are one way of optimising self-management and support for goal attainment. This can include support messages or reminders using electronic communication such as short message service (SMS) or email, as well as in-app messaging. Although SMS and internet-based programs have been trialled in several populations [15], their use in survivors of stroke is rare and limited to medication adherence or to a subgroup of those with hypertension or depression [14, 16, 17]. There is a need to build the evidence base on comprehensive eHealth messaging to enhance support for recovery, secondary prevention and self-efficacy following stroke.

In 2016, we developed an innovative multicomponent intervention comprised of standardised person-centred goal-setting and an aligned electronic self-management support system [18]. This intervention was specifically designed for people with stroke who are discharged home from the hospital. The system includes an online database to capture participant characteristics with automatic integration of patient characteristics, stroke recovery and prevention goals into a purpose-built iVERVE (inspiring Virtual Enabled Resources following Vascular Events) messaging system. The platform has > 1200 electronic messages that were developed using evidence-based behaviour change theory, clinical guidelines and independent review [18]. This new intervention required testing for feasibility and acceptability in people with stroke before application in a fully powered effectiveness trial.

## Aims

To assess the feasibility, acceptability and potential effectiveness of iVERVE as part of a pilot randomised controlled trial, among a convenience sample of survivors of stroke. Subsequently, this evidence would be used to inform the design of future trials.

## Methods

### Study design

**Fig. 1:**
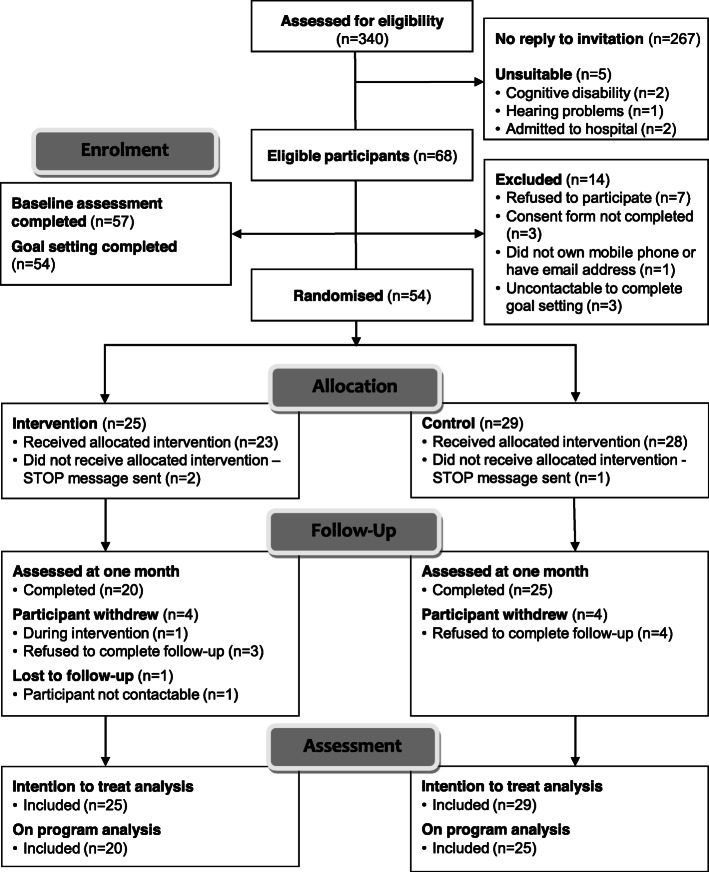
CONSORT diagram showing flow-through study for participants assessed for eligibility

We conducted, a prospective, two-group randomised (1:1) controlled pilot trial using a mixed-methods PROBE (prospective randomized, open-label, blinded-endpoint) design with an active control (Fig. 1). The trial was conducted in Melbourne, Australia.

The trial had an embedded, computer-generated randomisation process (schedule developed by an independent researcher otherwise uninvolved in the trial). Baseline and follow-up assessments were completed by an assessor who was blind to group allocation. A participant satisfaction survey was also completed. All participant trial data were entered into the Research Electronic Data Capture (REDCap) electronic data capture tool [19] hosted at Monash University. REDCap is a secure, web-based software platform designed to support data capture for research studies and has a range of features including interoperability with external sources [19].

The trial was retrospectively registered with Australian New Zealand Clinical Trials Registry: ACTRN12618001519246, registered on 11 September 2018. The Monash University Human Research Ethics committee (CF16/1920 - 2016000979) approved the study before data collection began. We report our methods and results in compliance with the CONSORT 2010 Statement, including the extension for randomised pilot and feasibility trials [20].

### Patient and public involvement

Public involvement was obtained in two ways. First, survivors of stroke and advocates from the Stroke Foundation were included on the advisory committee as part of overseeing the development of the intervention [18]. Second, we obtained feedback from survivors of stroke who were participants in the study during the conduct of this pilot trial. We will also disseminate a lay summary of the study results on our project-specific website http://recaps.com.au/ and provide this report to participants on request.

### Participant recruitment and randomisation

Participants for this study were recruited from the Australian Stroke Clinical Registry (AuSCR). The AuSCR is a national clinical quality registry of people with acute stroke or transient ischaemic attack. It is designed to monitor and improve the quality of acute stroke care [21]. Participants were included if they (1) agreed to participate in future research as described in their AuSCR 90–180-day follow-up survey, (2) were aged at least 18 years, (3) had been discharged from the hospital within 6–12 months of their last registered admission for stroke, (4) were living in the community (but not in residential aged care facilities), (5) were located within 50 km of Monash University (Monash Health, Clayton campus), and (6) had English as their first language or did not require an interpreter.

On behalf of the researchers, the AuSCR Office staff mailed an invitation pack that included a consent form and a pre-enrolment survey to potentially eligible participants. The reply-paid forms were returned to Monash University by interested participants. Subsequently, for those who completed the consent form and who confirmed their eligibility based on their responses to the pre-survey, an Outcome Assessor completed their baseline (*T*_0_) interviews by telephone. The blinded Outcome Assessor also completed follow-up calls after the 4-week program was completed (*T*_1_) (Fig. 1). Once the baseline assessment had been completed and before randomisation, a separate telephone interview was conducted by a clinician-researcher to help participants set 2–3 stroke recovery and prevention goals that were likely to be attainable within the 4-week timeframe of the intervention. The clinician-researcher was a qualified allied health professional with expert skills in setting recovery-focused health goals with patients after stroke. This was done using a project-specific standardised goal-setting template and procedure (see below) [18]. To ensure standardisation in study procedures, Outcome Assessors and clinician-researchers received relevant training by members of the investigator team.

Participants were randomly allocated 1:1 to the control or the intervention group by an independent researcher. Randomisation was stratified by age (< 65, 65+ years) and modified Rankin Scale score [0–2 (no symptoms at all or no significant disability despite symptoms), 3–5 (slight disability, moderate disability or moderately severe disability)] [22].

#### Sample size

We sought to recruit a maximum of 50 patients per group for this pragmatic feasibility (pilot) trial consistent with recommendations in the literature and other studies in this field [23–27].

### Control

Participants in the control group received their usual care in the community and to avoid unblinding to group allocation received goal-setting assistance for 2–3 goals and received 2–3 administrative messages over a 4-week period. This included a recommendation to access the Stroke Foundation (Australia) website for information about stroke through bit.ly/2tjtyhE, or phoning the Strokeline.

### Intervention

In addition to the goal-setting assistance received by the control group, the intervention group also received a comprehensive post-discharge support eHealth program that was delivered via the iVERVE system [18]. This system was developed by an interdisciplinary team including communication engineers, clinicians, public health researchers and consumers, with independent review of messages. It enables the programming of electronic support and educational messages aligned to nominated goals that cover the four domains of the International Classification of Function (ICF) developed by the World Health Organization [28]. Messages were created, based on behaviour change theory (including social cognitive theory, Information-Motivational-Behavioural theory and operant condition) and behaviour change techniques [18], under five main categories: administration and general motivation; secondary prevention; health/body function, activities and participation (combined ICF categories); and environment. The message bank included over 1200 messages. Participants in the intervention group received daily support messages matched to their personal recovery and prevention goals and level of functional ability. The messages could be tailored and delivered via SMS or email and were personalised (by name) and individualised, and some messages contained hyperlinks to trusted websites. Two-way communication was possible, e.g. ‘STOP’ messages could be received from participants’ telecommunications. The number of messages received did not exceed one per day and was dependent on the number of goals set. The intervention group also received one or two administrative or general motivational message per week.

### Outcomes

*Primary outcomes* for this study were defined as the number of participants who completed the trial, the number of goals attained and the number of participants satisfied with the program. Goal attainment was measured using Goal Attainment Scaling (GAS) [29]. The GAS is an established quantitative method that offers an individualised evaluation of non-linear constructs (i.e. patient-centred goals) based on mathematical principals to permit generalisability for use in group comparisons [30]. Within GAS, progress towards goal achievement is measured with reference to the goal and scored on a 5-point scale from − 2 (no progress has been made or performance worse than baseline) to + 2 (performance greatly exceeded expectation). A zero score indicates achievement of the expected level of performance, as indicated in the goal statement [29]. To promote reliability and consistency, a clinical member of the research team who was not part of the goal-setting or evaluation team pre-populated the expected GAS levels of performance (− 2 through + 2) [31].

*Secondary outcomes* were intervention dose (number of SMS/email messages successfully sent), costs of maintaining the iVERVE interface and sending messages, and satisfaction with the content of eHealth messages. Other secondary outcomes include self-management (measured with the validated Health Education Impact Questionnaire [heiQ]) [32] which covers constructs related to enhancing self-efficacy as measured through self-management practices such as health-directed behaviours, self-monitoring and insight and skill acquisition to cope with symptoms and health problems [33]. Higher scores characterise better skills [32]. We have previously used this outcome in a trial of self-management programs for survivors of stroke [34]. Given the large number of people after stroke who self-report anxiety and depression [35], we also measured emotional status (Hospital Anxiety and Depression Scale [HADS]) with lower scores indicating fewer symptoms [36]. Health-related quality of life is measured by the EuroQoL-5 dimension-3 (EQ-5D-3L) level instrument and the visual analogue scale (VAS; 0–100 with higher scores indicating better self-reported health status) [37]. We also collected information on participation using the Nottingham Extended Activities of Daily Living (NEADL; 0–66 with higher scores indicating independence with everyday activities) Scale [38]. All of these health rating scales have previously been used in stroke studies and validated for use in stroke [38–40].

Outcomes were measured at baseline (*T*_0_) for health status and after the intervention (*T*_1_ after week 5).

The modified Rankin Scale [22] was used to assess the degree of disability or dependence at baseline to assist with the allocation of support messages based on the level of disability.

### Feasibility assessment and process evaluation

Feasibility was assessed against the variables of recruitment of participants, dose of the intervention delivered, message transmission failures, completeness of baseline and outcome data, and respondent burden, including retention. We triangulated sources of information including administrative data, calls or email to a help-desk contact person and log-records of electronic messaging activity. Log-records were also used to calculate the costs of calls.

Recruitment proportion was determined by calculating the number of consented participants as a proportion of the eligible population and retention of consented participants. Characteristics of the non-responders were obtained from the AuSCR. *Intervention fidelity* was partly assessed by examining the number of text messages sent and those that failed to send; measures of acceptability included the number of respondents who sent ‘STOP’ texts and the reasons for the ‘STOP’ texts (also considered a perceived measure of burden). Feasibility of *measurement* and the burden of data collection were determined by assessing the number of clinical outcomes completed and recording the duration of the goal-setting interviews. In our prior research of a group-based self-management intervention, we have found that survivors of stroke, despite the different types of impairments, complete this type of health outcome battery with acceptable response rates [41].

### Satisfaction

A survey was sent to participants 1–2 weeks post-intervention to assess their feedback with various aspects of the intervention including goal-setting. This included closed questions and open text fields to describe the perceived benefits of the program, willingness to continue with the program or likelihood of recommending it to other survivors of stroke. Participants in the intervention group were asked additional questions to elicit their perceived benefits of receiving electronic health support. These included questions about the type and frequency of electronic messages, appropriateness of message content and perceptions about the adequacy of the length of support. A focus group was also undertaken with a sample of those in the intervention group [42] (data not reported).

### Data analysis

Descriptive statistics were used to compare the participants’ characteristics by group allocation (intervention or control) and also with non-responders (those who did not reply to the invitation to participate, or were deemed ineligible after initial screening, or were uncontactable after initially responding, or chose not to participate once they learnt about the project). All outcome measures were analysed using an intention-to-treat analysis. Descriptive statistics were calculated for all variables over the two time points (*T*_0_, *T*_1_). Due to the skewed distribution of continuous outcomes, differences between the groups were reported as the median difference (95% CI). Median or logistic regression models were used with the outcome as the dependent variable (at the 4-week follow-up) and baseline scores entered as covariates. Independent variables, including group allocation, were entered into the regression. In the sensitivity analyses using median regression, bootstrap estimates were computed for 1000 replicates for 25th, 50th and 75th quantiles to calculate the change at 4 weeks relative to baseline measurements for assessing potential within-group differences. Where relevant, statistical significance was set at *p* < 0.05 (two-sided). Open-ended responses from the satisfaction surveys were analysed using inductive thematic analysis [43]. Closed questions were summarised descriptively.

For GAS, the GAS-T method was used [29]. Each goal was given a score over 5 levels ranging from − 2 to + 2. Goals were also weighted based on relative importance to the patient, as well as difficulty as perceived by the clinician involved in the collaborative goal-setting, with each graded on a scale of 0 (not at all difficult) to 3 (very difficult) [44]. Individual scores were then combined to provide an overall goal attainment score. The GAS-transformed normally distributed score (i.e. *T* score) was calculated for each intervention group by goal category with a mean of 50 (SD 10) as the reference [29]. The *T* score was categorised by attainment of goal status (e.g. goal not attained = *T* score < 50 and goal often attained = *T* score ≥ 50).

All analyses were undertaken using Stata/SE 15.01 (StataCorp 2017).

## Results

### Feasibility outcomes: participation, retention and data completeness

Recruitment for this study began on 28 March 2017, and the final baseline assessments were completed on 8 August 2017, to ensure our pilot study would be completed in 2017. The completion of outcome assessments occurred between 29 June and 14 November 2017.

During the screening and recruitment phase, 340 eligible AuSCR registrants were invited to participate. Of the 73 who returned a consent form and were subsequently assessed for eligibility, five were deemed unsuitable for this study and did not proceed to the next stage (i.e. two participants had cognitive disabilities, two had been admitted to hospital and one had hearing problems making communication for telephone outcome assessments infeasible). Compared with the non-responders, participants were more likely to be younger and born in Australia (not statistically significant).

Among the 68 remaining candidates for inclusion in this study, 57 (84%) completed the baseline assessment. These participants were living at home ~ 12–24 months after their initial stroke event. However, eleven participants (16%) did not complete the baseline assessment for the following reasons: seven declined to participate, three did not return a consent form and one participant did not own a mobile phone or have an email address. At the goal-setting interview stage, three participants were uncontactable, leaving 54 (95%) participants who were randomised (Fig. 1). Twenty-five were allocated to the intervention group (median age 69 years; 40% female) and 29 were allocated to the control group (mean age 68 years; 38% female). Baseline characteristics were similar between the groups (Table 1). The demographics of the participants who completed all baseline measures and set goals were similar to individuals classified as non-responders (*n* = 286; ~ 40% female; Additional file 1: Table 1). The overall retention in the study was 45/54 (83%) (Fig. 1).

**Table 1:** Characteristics of participants

Baseline characteristics	Control, ***n***/***N*** (%), ***N*** = 29^**d**^	Intervention, ***n***/***N*** (%), ***N*** = 25^**d**^
**Demographics**		
Age, mean (SD)	68 (10)	69 (11)
Female	11/29 (38)	10/25 (40)
Australian	20/29 (69)	20/25 (80)
Married/with partner	20/29 (69)	12/25 (48)
Live independently	7/28 (25)	9/25 (36)
Own home or unit	25/27 (93)	22/24 (92)
Retired	17/27 (63)	14/24 (58)
University educated	9/28 (32)	8/24 (33)
**Preference for electronic messages**		
Preferred SMS communication	12/29 (41)	14/25 (56)
**Use of health services and private health insurance status**		
Private health insurance	19/29 (66)	18/25 (72)
Hospital and extras cover	13/19 (68)	13/18 (72)
Use community services	3/29 (10)	8/25 (32)
Allied care services in last 4 weeks	5/29 (17)	5/23 (22)
**Self-reported medical history**		
Hypercholesterolaemia	16/28 (57)	15/24 (63)
Heart attack	4/26 (15)	5/24 (21)
Atrial fibrillation	7/26 (27)	10/24 (42)
Hypertension	21/29 (72)	15/24 (63)
Sleep apnoea	3/26 (12)	4/24 (17)
Respiratory problems	3/26 (12)	3/24 (13)
Diabetes	6/28 (21)	4/24 (17)
Arthritis	11/25 (44)	11/24 (46)
Depression	4/26 (15)	6/24 (25)
Anxiety	5/26 (19)	3/24 (13)
Cancer	4/26 (15)	1/23 (4)
Other illness	3/23 (13)	6/23 (26)
**Lifestyle characteristics**		
Smoking^a^		
Current smoker	1/27 (4)	1/25 (4)
Past smoker	14/27 (52)	9/25 (36)
Physically active^b^	9/16 (56)	15/17 (88)*
Alcohol consumption	21/28 (75)	17/25 (68)
Risky drinking^c^	4/21 (19)	4/16 (25)
Healthy eating		
Advised to change diet	6/29 (21)	9/25 (36)
> 5 servings of vegetables daily	1/29 (3)	2/23 (9)
> 2 servings of fruit daily	13/29 (45)	15/23 (65)
**Independent**	20/29 (69)	19/25 (76)

Independent: none to slight disability classified using the modified Rankin Scale 0–1 scores

*SD* standard deviation, *SMS* short message service

^a^Self-report of current smoking status

^b^Undertaking > 20 min of vigorous intensity physical activity ≥ 3 times per week

^c^Risky drinking is defined as ≥ 2 drinks per day for women and ≥ 4 drinks per day for men

^d^For variables with missing observations, the denominator is less than the total number for each intervention group

*Statistical significance *p* < 0.05

The median minutes (interquartile range) taken to complete assessments was 22 (15; 31) at baseline, 29 (22; 36) at follow-up and 35 (30; 45) for setting a maximum of three goals. At *T*_1_, the outcome assessment was unable to be collected from nine participants, with eight withdrawing (4 intervention and 4 control). One intervention participant was uncontactable. Outcome measures were able to be collected from 45 participants across two time points with > 95% complete data. Median time from start to completion of follow-up was 35 days.

### Goal attainment

Based on the raw GAS scaling scores, overall, 92% of participants scored − 1 or above indicating some progress towards achieving their goals during the 4-week period. For the whole sample, goal attainment using the mean GAS *T* scores across all goals was not achieved (mean 49, SD 13; Table 2). Among the intervention group, goal attainment was achieved in three out of the four goal categories: health/body function, activities, and participation and environment, whereas goal attainment in the control group was only achieved for goals relating to environment tasks.

**Table 2:** Goal Attainment Scaling (GAS) at follow-up for intervention and control groups

Type of goals	Overall, mean ***T*** score (SD), ***Ν*** = 42	Control, mean ***T*** score (SD), ***N*** = 23	Intervention, mean ***T*** score (SD), ***N*** = 19
***All goals***	49 (13)	48 (11)	49 (15)
Secondary prevention	45 (13)	45 (11)	44 (15)
Health/body function	50 (13)	48 (14)	53 (12)
Activities and participation	53 (17)	48 (9)	53 (17)
Environment	52 (14)	50 (4)	53 (20)

*SD* standard deviation, *GAS* Goal Attainment Scaling scores: goal not attained = T score < 50, goal often attained = T score ≥ 50

### Participant views

The survey was completed by 27/54 (50%) of participants (13 intervention: 52%; 14 control: 48%). Most participants in both groups stated they would be happy to take part in a similar project in the future (intervention 69%, control 71%) and would recommend it to other people with stroke (intervention 85%, control 79%).

### Satisfaction with goal-setting procedures

Over 85% of participants in both groups reported that the goal-setting form was helpful in developing their goals. Both groups agreed that the clinicians were helpful in developing their goals (intervention 92%, control 72%, *p* = 0.16).

### Perceived benefit of the electronic health support (intervention group only)

**Fig. 2:**
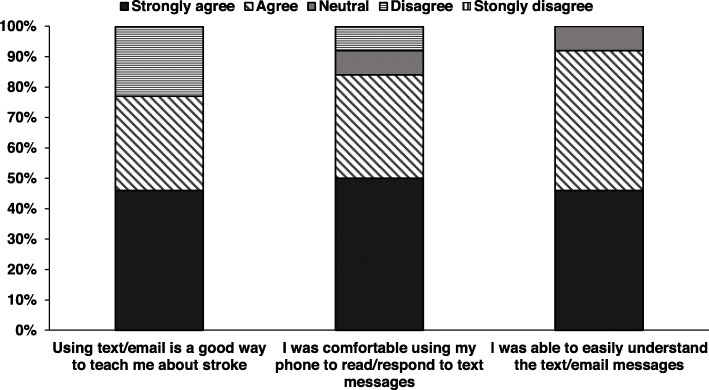
Acceptability of the electronic support messages—intervention group (*n* = 13)

**Fig. 3:**
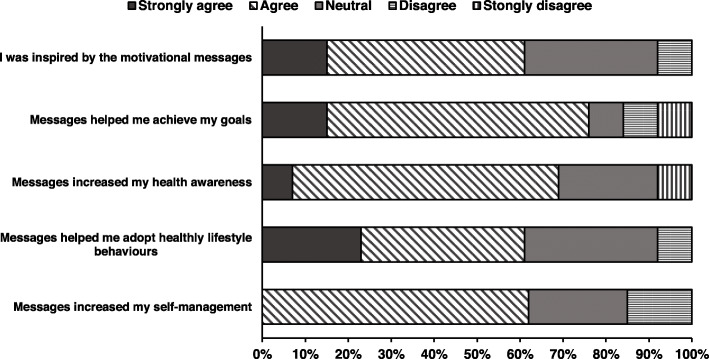
Perceived benefits of electronic messages received by participants—intervention group (*n* = 13)

No unintended harms or effects were reported. Most participants believed that text or email messages helped them achieve their goals (77%) and were a good way to receive education about stroke (Fig. 2). Participants were comfortable accessing their mobile phone or computer to read and respond to SMS or email messages and felt that the iVERVE messages were easy to understand. This group perceived many benefits to the electronic support messages (Fig. 3). Eleven of the 13 participants in the intervention group who responded to the survey considered the frequency of SMS/email messages received was appropriate. Overall, the messages were considered easy to receive and read (85%). However, only 54% (*n* = 7) reported that they understood how to access further information from the web links provided within the messages.

### Potential benefits for self-management or health outcomes

Among participants in the intervention group, we noted several indicative improvements in different domains of the heiQ at 4 weeks (Additional file 1: Table 2). For example, in the univariable analyses in the intervention group, the median score for constructive attitudes and approaches was 5.8 at baseline and 6.0 at follow-up. The median change at 4 weeks relative to baseline measurements was 1.25 (95% CI 0.3, 2.2). In the sensitivity analyses, similar results in the intervention group were found at the 50th quantile compared with 25th and 75th quantiles for the median change at 4 weeks relative to baseline measurements (Additional file 1: Table 3). After adjusting for baseline measures, we found preliminary evidence of potential improvements for most of the self-management domains, in favour of the intervention group compared with the control group (Table 3). For example, for social integration and support, there a was positive increase (coefficient 0.34) in this domain at follow-up after adjustment for the effect of the intervention on outcomes. We also noted potential improvements to some quality-of-life dimensions (EQ-5D-3L): self-care, usual activities, and pain or discomfort.

**Table 3:** Between-group differences: intervention minus control (adjusted analyses of the potential effect of the intervention in self-management or health outcomes)

Outcome	Effect estimate for complete case analysis
***heiQ (self-management)***	***β*** **coefficient (95% CI)**^**a**^
Positive and active engagement in life	0.07 (− 0.61, 0.74)
Health directed behaviour	0.25 (− 0.50, 1.00)
Skill and technique acquisition	0.20 (− 0.33, 0.73)
Constructive attitudes and approaches	0.00 (− 0.62, 0.62)
Self-monitoring and insight	0.29 (− 0.18, 0.75)
Health service navigation	0.26 (− 0.20, 0.72)
Social integration and support	0.34 (− 0.14, 0.83)
Emotional wellbeing	− 0.08 (− 0.80, 0.63)
***Emotional status***	***β*** **coefficient (95% CI)**^**a**^
HADS: depression	0.00 (− 1.36, 1.36)
HADS: anxiety	0.33 (− 1.06, 1.73)
***NEADL (participation)***	
Mobility	− 0.89 (− 3.18, 1.41)
Kitchen	0.00 (− 0.37, 0.37)
Domestic	0.00 (− 0.96, 0.96)
Leisure	0.00 (− 2.20, 2.20)
***Visual analogue scale (EQ-5D)***	− 1.25 (− 7.28, 4.78)
***EQ-5D (quality of life)***	**OR (95% CI)**^**b**^
Mobility	1.19 (0.26, 5.40)
Self-care	0.18 (0.02, 2.56)
Usual activities	0.56 (0.12, 2.62)
Pain or discomfort	0.57 (0.12, 2.74)
Anxiety or depression	1.54 (0.19, 12.45)

*CI* confidence interval, *heiQ* Health Education Impact Questionnaire, missing individual options were replaced with the average score of the specific dimension, *HADS*, Hospital Anxiety and Depression Scale, *NEADL* Nottingham Extended Activities of Daily Living Scale, missing individual options were replaced with the value 3 (‘On your own easily’), *EQ-5D* EuroQol health-related quality of life five dimensions questionnaire and Visual Analogue Scale, *OR* odds ratio

^a^Median regression

^b^Logistic regression

### Program costs and dose

Overall, 824 electronic messages (446 SMS; 378 emails) were sent during the intervention period (657 intervention; 167 control). The average number of messages sent was 15 for the intervention participants and 3 for control participants. There were no message failures and no calls to the help desk to report problems with the messages. In terms of returned messages, there were two ‘STOP’ requests and one request for ‘no more contact’ from the participants (2 intervention; 1 control). The total cost for delivering the 824 messages was 39 Australian dollars (AUD39, or 4.7 cents per message sent).

## Discussion

We provide preliminary evidence that the use of electronic messaging to support comprehensive person-centred goal achievement after stroke is feasible and has potential in terms of effectiveness. In particular, we found that our comprehensive iVERVE intervention was acceptable to people with stroke in providing support and health information on a broad range of recovery issues. Another aim of this pilot study was to establish the feasibility of our goal-setting method and acceptance of a purpose-built iVERVE messaging system. We were encouraged that a larger proportion of intervention participants believed that the health professionals had been helpful in facilitating development of person-centred goals across a range of domains since they received messages tailored to these goals, compared to the control group. Goal attainment differences between the groups in this sample of people with chronic stroke who trialled the intervention for 4 weeks were promising. Median *T* scores over 50, indicating goals often obtained, were noted for the intervention group for health/body function, activities and participation and environment, but not the control group. Limitations of using the GAS include the small sample size and short intervention period. It was also reassuring to find preliminary evidence of improvements for most of the self-management domains and several quality-of-life domains in favour of the intervention group compared with the control group.

Few studies have been conducted which include goal-setting with eHealth support. In the study by Wan et al., people with ischaemic stroke were randomised to control, or a goal-setting intervention without eHealth support messages [45]. Participants in this latter study were assisted in establishing secondary prevention goals during a telephone call 1 week after discharge. Two additional follow-up calls (1 and 3 months post-discharge) were conducted by the stroke nurse to discuss progress towards their goals. The intervention was feasible and led to improved medication adherence at 6 months after discharge from the hospital [45], but there were no other differences between the groups on other health behaviour categories or disability outcomes. The authors concluded that there was a need for more effective intervention strategies with increased contact, to help participants reach guideline-recommended targets [45]. Subsequently, these authors conducted a multicomponent intervention study in people with hypertension and ischaemic stroke to improve health behaviours and blood pressure control [14]. The intervention, provided over 4 weeks, comprised face-to-face and telephone health belief education, a participant calendar handbook and weekly automated short message services. With the exception of smoking and alcohol consumption, health behaviours targeting other risk factors and blood pressure control improved [14], which lends support for incorporating secondary prevention as part of iVERVE. However, our approach is more comprehensive than this prior work and acknowledges the diverse impact of stroke on many aspects of life, as well as the need to enable support for secondary prevention.

Strengths of this study were the overall high level of acceptability for the intervention, the lack of message failures and the demonstrated ability to recruit participants from an established national clinical quality registry which created recruitment efficiencies for a pilot feasibility trial. Well-designed randomised controlled trials (RCTs) provide the highest level of quality for assessing the efficacy of interventions [46]. However, achieving recruitment targets can be difficult, leading to extended study timeframes, additional costs or wastage of valuable research money when studies fail to reach targets [47]. As an alternate strategy, clinical registries may provide vehicles to facilitate recruitment for clinical trials, particularly trials recruiting community-dwelling stroke survivors. We found that the use of the AuSCR was a cost-effective way to recruit for the current study, since between 60 and 64% of registrants at the 90–180-day follow-up indicate their willingness to be contacted for research [48]. Compared to those unwilling to be contacted, these registrants were younger and more often male [48]. The current study provides one of the first examples of leveraging this registry infrastructure to conduct a clinical trial in stroke. Clinical registries may also provide an efficient mechanism to monitor uptake of new evidence for different contexts and settings and can be complementary to conducting clinical trials [49, 50]. If our messaging intervention is found to be effective in a phase III RCT, it could be routinely monitored through national registry infrastructure such as AuSCR.

The limitations of this pilot study include the 4-week time frame for testing the intervention and the inclusion of participants who had their stroke over 1 year before receiving the intervention. For pragmatic reasons, we piloted the intervention for only 4 weeks (although the intervention has been designed as a 12-week program) since 4 weeks was considered sufficient to provide us with the information required for a future phase II trial regarding program acceptability, system integrity and feedback on when and how it might best be used from the perspective of someone living with stroke. Consistency in the delivery of the intervention was monitored by the number of messages that were sent, and if any failed to send. We did not collect information on what actions were undertaken to achieve goals. We acknowledged that the findings reported in this paper may not equate to those obtained if the full intervention had been tested. Overall, few (17%) participants withdrew or were uncontactable. However, we are unable to comment on whether a greater proportion may withdraw from a larger/longer study or one in which patients immediately discharged from the hospital are included. A process evaluation to examine responder burden and retention rates from a longer intervention period and the delivery of a greater number of text messages will be considered in a future study.

There is currently little evidence to guide best practice for supporting people with stroke in the community immediately post-discharge [51]. In the era of widely available technology used by most of the population at all ages, low cost, scalable electronic support to increase self-management with professional facilitation of goal-setting may improve the transition to home and reduce hospital readmissions. In conducting this pilot trial, we have obtained important information to finalise various aspects of the intervention and the design of phase II/III trials in people with acute stroke discharged directly to home. Specifically, the data from this initial pilot study and our recently completed phase II study in acute stroke has informed the calculation of the sample size for our phase III study (*N* = 890) that has been funded by the National Health and Medical Research Council (1162596) to trial the intervention in people with stroke directly discharged from acute hospitals. This Recovery-focused Community support to Avoid readmissions and improve Participation after Stroke (ReCAPS) trial has recently commenced (ACTRN 12618001468213).

## Conclusions

With improved survival after stroke and recognised unmet needs of those living in the community, our comprehensive iVERVE intervention holds promise for addressing a range of educational and self-management issues faced everyday by survivors of stroke. Trials to determine the potential effectiveness and cost-effectiveness in acute stroke are underway.

## Supplementary Information

**Additional file 1:** **Table 1.** Characteristics of participants and non-responders. **Table 2.** Within group differences in self-management or health outcomes (T_1_-T_0_), for intervention and control groups. **Table 3.** Sensitivity analysis for within group differences in outcomes (T_1_-T_0_), for intervention and control groups

## Data Availability

Data from this clinical trial will be available upon reasonable request to the corresponding author following completion and publication of the phase III trial.

## Abbreviations

ANZCTR: Australian New Zealand Clinical Trials Registry
AUD: Australian dollars
AuSCR: Australian Stroke Clinical Registry
EQ-5D-3L: EuroQoL-5 dimension-3 level health-related quality of life questionnaire
GAS: Goal Attainment Scaling
HADS: Hospital Anxiety and Depression Scale
heiQ: Health Education Impact Questionnaire
ICF: International Classification of Function
iVERVE: Inspiring Virtual Enabled Resources following Vascular Events
NEADL: Nottingham Extended Activities of Daily Living
NHMRC: National Health and Medical Research Council
PROBE: Prospective randomized, open-label, blinded-endpoint
ReCAPs: Recovery-focused Community support to Avoid readmissions and improve Participation after Stroke
RCT: Randomised controlled trial
REDCap: Research Electronic Data Capture
SMS: Short message service
VAS: Visual analogue scale
