# Supplementary material for: Pilot randomised clinical trial of an eHealth, self-management support intervention (iVERVE) for stroke: feasibility assessment in survivors 12–24 months post-event
Source: Pilot Feasibility Stud. 2020 Nov 7;6:172. doi: 10.1186/s40814-020-00706-x (PMC7648386; doi:10.1186/s40814-020-00706-x)
Supplement: Supplementary file 1 — Additional file 1: Table 1. Characteristics of participants and non-responders. Table 2. Within group differences in self-management or health outcomes (T1-T0), for intervention and control groups. Table 3. Sensitivity analysis for within group differences in outcomes (T1-T0), for intervention and control groups [file 40814_2020_706_MOESM1_ESM.docx]

**ADDITIONAL FILE 1**

**Pilot randomised clinical trial of an ehealth, self-management support intervention (iVERVE) for stroke survivors: feasibility assessment 12-24-months post event**

Dominique A Cadilhac PhD^1,2^, Nadine E Andrew PhD^1,3^, Doreen Busingye PhD^1,4^, Jan D Cameron PhD^1,5^, Amanda G Thrift PhD^1^, Tara Purvis^1^, Jonathan C Li^6^, Ian Kneebone PsychD^7^, Vincent Thijs MD, PhD^2,8^, Maree L Hackett PhD^9,10 *^, Natasha A Lannin PhD^11,12^, Monique F Kilkenny PhD^1,2^ on behalf of the ReCAPS investigators

1. Stroke and Ageing Research, Department of Medicine, School of Clinical Sciences at Monash Health, Monash University, Clayton, VIC, Australia
2. Florey Institute of Neuroscience and Mental Health, the University of Melbourne Heidelberg, VIC, Australia
3. Department of Medicine, Peninsula Clinical School, Central Clinical School, Monash University, Frankston, VIC, Australia
4. NPS MedicineWise, Sydney, NSW, Australia
5. School of Nursing and Midwifery, Monash University, Clayton, VIC, Australia
6. Faculty of Engineering, Monash University, Clayton, VIC, Australia
7. Discipline of Clinical Psychology, Graduate School of Health, University of Technology Sydney, Ultimo, NSW, Australia
8. Department of Medicine, Austin Health, Heidelberg, VIC, Australia
9. The George Institute for Global Health, Faculty of Medicine, University of New South Wales, Sydney, NSW, Australia
10. Faculty of Health and Wellbeing, The University of Central Lancashire, Preston, United Kingdom
11. Department of Neurosciences, Central Clinical School, Monash University, Melbourne, VIC, Australia
12. Department of Allied Health (Occupational Therapy), Alfred Health, Melbourne, VIC, Australia

* Faculty of Health and Wellbeing, The University of Central Lancashire, Preston, United Kingdom

**Corresponding author:**

Professor Dominique A. Cadilhac ORCID: 0000-0001-8162-682X

Stroke and Ageing Research,

School of Clinical Sciences at Monash Health,

Monash University,

Level 3 Hudson Institute Building,

27-31 Wright Street, Clayton VIC 3168

Phone: +61 (3) 8572 2657

Email: dominique.cadilhac@monash.edu

**Key words**: stroke, ehealth, feasibility studies, healthcare technology

**Table I: Characteristics of participants and non-responders^#^**

| **Baseline characteristics** | **Participants**  **n (%)**  **N=54** | **Non-responders^#^**  **n (%)**  **N=286** |
| --- | --- | --- |
| Age, mean (SD) | 65 (14) | 68 (13) |
| Female | 21 (39) | 115 (40) |
| Australian born | 39 (72) | 182 (64) |
| In-hospital stroke | 2 (4) | 7 (3) |
| Previous stroke | 8 (15) | 42 (15) |
| Ischaemic stroke | 48 (89) | 246 (86) |
| Able to walk on admission | 21 (42) | 106 (39) |
| Discharge home from acute | 26 (49) | 138 (49) |

^#^Non-responders include those who did not reply to the invitation to participate, were deemed ineligible after the pre-screening survey, were uncontactable after initially responding, or chose not to participate once they learnt more about the project

**Table II: Within group differences in self-management or health outcomes (T_1_-T_0_), for intervention and control groups**

|  | **Control** | | | **Intervention** | | |
| --- | --- | --- | --- | --- | --- | --- |
|  | **Baseline T_0_**  **N=29** | **Follow-up T_1_**  **N=25** | **Median Difference^a^**  **(95% CI)**  **T_1_- T_0_** | **Baseline T_0_**  **N=25** | **Follow-up T_1_**  **N=20** | **Median Difference^a^ (95% CI)**  **T_1_- T_0_** |
| ***heiQ (self-management)*** | **Median(Q1, Q3)** | **Median(Q1, Q3)** |  | **Median(Q1, Q3)** | **Median(Q1, Q3)** |  |
| Positive & active engagement in life | 5.2 (4.8, 5.6) | 5.4 (4.8, 6.0) | 0.73 (0.2, 1.3) | 5.6 (4.6, 6.0) | 5.6 (5.0, 6.0) | 1.00 (0.6, 1.4) |
| Health directed behaviour | 4.8 (4.0, 6.0) | 4.5 (3.5, 5.5) | 0.65 (0.4, 0.9) | 5.5 (5.0, 5.8) | 5.3 (4.5, 6.0) | 1.05 (0.7, 1.4) |
| Skill and technique acquisition | 5.0 (4.8, 5.4) | 5.2 (5.0, 5.6) | -0.20 (-1.1, 0.7) | 5.3 (5.0, 5.6) | 5.6 (5.0, 6.0) | 0.33 (-0.0, 0.7) |
| Constructive attitudes and approaches | 5.2 (5.0, 5.6) | 5.1 (5.0, 5.8) | 0.4 (-0.4, 1.2) | 5.8 (5.2, 6.0) | 6.0 (5.0, 6.0) | 1.25 (0.3, 2.2) |
| Self-monitoring and insight | 5.1 (5.0, 5.7) | 5.2 (5.0, 5.9) | 0.71 (-0.2, 1.7) | 5.6 (5.1, 5.9) | 5.4 (5.1, 6.0) | 0.50 (-0.6, 1.7) |
| Health service navigation | 5.8 (5.0, 6.0) | 5.5 (5.0, 5.8) | 0.50 (-0.0, 1.0) | 5.8 (5.4, 6.0) | 6 (5.0, 6.0) | 0.71 (-0.0, 1.5) |
| Social integration and support | 5 (4.6, 6.0) | 5.1 (5.0, 6.0) | 0.64 (0.2, 1.0) | 5.4 (4.8, 6.0) | 5.2 (5.0, 6.0) | 0.67 (0.2, 1.1) |
| Emotional wellbeing | 2 (1.3, 3.7) | 2 (1.8, 2.5) | 0.58 (0.1, 1.1) | 1.5 (1, 3.2) | 2 (1.0, 2.5) | 0.95 (0.3, 1.6) |
| ***Emotional status*** |  |  |  |  |  |  |
| HADS: Depression | 2 (1, 5.5)^ | 3 (1, 4)^ | 0.56 (0.4, 0.7) | 2 (1, 4)^ | 2 (1, 7) | 0.89 (0.3, 1.5) |
| HADS: Anxiety | 4 (2, 6) | 4 (2, 5)^ | 0.43 (0.1, 0.8) | 5.5 (3, 7)^ | 5 (1.5, 7) | 0.75 (0.4, 1.1) |
| ***NEADL*** |  |  |  |  |  |  |
| Mobility | 18 (15, 18) | 15 (14, 17) | 0.38 (-0.2, 1.0) | 17 (14, 18) | 15 (12, 18) | 1.06 (0.4, 1.7) |
| Kitchen | 15 (14, 15) | 15 (15, 15) | 0.00 (-1.0, 1.0) | 15 (15, 15) | 15 (15, 15) | 1.00 (0.5, 1.5) |
| Domestic | 15 (12, 15) | 13 (11, 15) | 1.00 (0.8, 1.2) | 15 (13, 15) | 15 (13, 15) | 0.92 (-0.1, 1.9) |
| Leisure | 17 (15, 18) | 15 (12, 18) | 0.43 (-0.2, 1.1) | 18 (15, 18) | 15 (13, 18) | 1.50 (0.8, 2.2) |
| ***Visual analogue scale (EQ-5D)*** | 80 (75, 90) | 80 (75, 90) | 0.71 (0.3 to 1.2) | 80 (70, 95) | 80 (73, 89) | 0.78 (0.4 to 1.1) |
| ***EQ-5D – some/moderate problems*** | **n (%)** | **n (%)** |  | **n (%)** | **n (%)** |  |
| Mobility | 8 (28) | 7 (28) | - | 11 (44) | 8 (40) | - |
| Self-care | 2 (7) | 4 (16) | - | 3 (12) | 1 (5) | - |
| Usual activities | 15 (52) | 10 (40) | - | 10 (40) | 5 (25) | - |
| Pain or discomfort | 11 (38) | 9 (36) | - | 11 (44) | 7 (35) | - |
| Anxiety or depression | 8 (28) | 5 (20) | - | 10 (40) | 7 (35) | - |

***Table II: Footnotes***

**CI:** Confidence interval; **Q1**: Quartile 1; **Q3**: Quartile 3; **^a^:** determined using median regression, bootstrap estimates were computed for 1000 replicates to calculate the median (50^th^ quantile) change at 4 weeks relative to baseline measurements for within group differences; **heiQ**: Health Education Impact Questionnaire, missing individual options were replaced with the average score of the specific dimension; **HADS**: Hospital Anxiety and Depression Scale; **NEADL**: Nottingham Extended Activities of Daily Living Scale, missing individual options were replaced with the value 3 (‘On your own easily’); **EQ-5D:** EuroQol health-related quality of life five dimensions questionnaire – number reporting some/moderate problems on each domain. **^:**1-2 cases missing.

**Table III: Sensitivity analysis for within group differences in outcomes (T_1_-T_0_), for intervention and control groups**

|  | **Control** | | | **Intervention** | | | |
| --- | --- | --- | --- | --- | --- | --- | --- |
| **Difference** | **25^th^ quantile^a^**  **(95% CI)**  **T_1_-T_0_** | **Median (50^th^) ^a^**  **(95% CI)**  **T_1_-T_0_** | **75^th^ quantile^a^**  **(95% CI)**  **T_1_-T_0_** | **25^th^ quantile^a^ (95% CI)**  **T_1_-T_0_** | **Median (50^th^)^a^ (95% CI)**  **T_1_-T_0_** | **75^th^ quantile^a^ (95% CI)**  **T_1_-T_0_** |  |
| ***heiQ*** |  |  |  |  |  |  |  |
| Positive & active engagement in life | 0.60 (-0.1, 1.3) | 0.73 (0.2, 1.3) | 0.50 (-0.1, 1.1) | 1.00 (-0.1, 2.1) | 1.00 (0.6, 1.4) | 0.64 (0.2, 1.0) |  |
| Health directed behaviour | 0.53 (0.1, 0.9) | 0.65 (0.4, 0.9) | 0.64 (0.3, 0.9) | 1.00 (0.3, 1.8) | 1.05 (0.7, 1.4) | 0.86 (0.2, 1.6) |  |
| Skill and technique acquisition | 0.33 (-0.7, 1.4) | -0.20 (-1.1, 0.7) | 0.09 (-0.6, 0.7) | 0.31 (-0.8, 1.4) | 0.33 (-0.0, 0.7) | 0.13 (-0.3, 0.5) |  |
| Constructive attitudes and approaches | 0.12 (-1.4, 1.7) | 0.4 (-0.4, 1.2) | 0.55 (0.1, 0.9) | 1.07 (0.0, 2.1) | 1.25 (0.3, 2.2) | 0.50 (-0.6, 1.6) |  |
| Self-monitoring and insight | 0.00 (-0.8, 0.8) | 0.71 (-0.2, 1.7) | 0.50 (-0.3, 1.3) | 0.50 (-1.5, 2.5) | 0.50 (-0.6, 1.7) | 0.35 (-0.3, 0.9) |  |
| Health service navigation | 0.00 (-0.5, 0.5) | 0.50 (-0.0, 1.0) | 0.38 (-0.1, 0.9) | 0.75 (-0.2, 1.7) | 0.71 (-0.0, 1.5) | 0.00 (-0.8, 0.8) |  |
| Social integration and support | 0.57 (0.2, 0.9) | 0.64 (0.2, 1.0) | 0.71 (0.3, 1.2) | 0.75 (0.0, 1.5) | 0.67 (0.2, 1.1) | 0.56 (0.2, 0.9) |  |
| Emotional wellbeing | 0.35 (-0.2, 0.9) | 0.58 (0.1, 1.1) | 0.58 (0.3, 0.9) | 0.41 (-0.2, 0.9) | 0.95 (0.3, 1.6) | 0.75 (0.1, 1.4) |  |
| ***Emotional status*** |  |  |  |  |  |  |  |
| HADS: Depression | 0.64 (0.5, 0.8) | 0.56 (0.4, 0.7) | 0.44 (0.2, 0.7) | 0.33 (-0.3, 0.9) | 0.89 (0.3, 1.5) | 1.00 (0.1, 1.9) |  |
| HADS: Anxiety | 0.60 (0.3, 0.9) | 0.43 (0.1, 0.8) | 0.50 (0.0, 0.9) | 0.71 (0.3, 1.1) | 0.75 (0.4, 1.1) | 1.00 (0.6, 1.4) |  |
| ***NEADL*** |  |  |  |  |  |  |  |
| Mobility | 0.63 (0.0, 1.3) | 0.38 (-0.2, 1.0) | 0.38 (-0.0, 08) | 0.88 (0.0, 1.7) | 1.06 (0.4, 1.7) | 0.70 (0.3, 1.1) |  |
| Kitchen | 0.75 (-0.7, 2.2) | 0.00 (-1.0, 1.0) | 0.00 (-0.6, 0.6) | 1.00 (0.0, 1.9) | 1.00 (0.5, 1.5) | 0.60 (0.1, 1.1) |  |
| Domestic | 0.83 (0.4, 1.3) | 1.00 (0.8, 1.2) | 0.75 (0.3, 1.1) | 1.25 (-0.3, 2.8) | 0.92 (-0.1, 1.9) | 0.00 (-1.0, 1.0) |  |
| Leisure | 0.88 (0.2, 1.6) | 0.43 (-0.2, 1.1) | 0.63 (0.2, 1.1) | 1.60 (0.9, 2.3) | 1.50 (0.8, 2.2) | 1.00 (-0.0, 2.0) |  |

**CI:** Confidence interval; **Q1**: Quartile 1; **Q3**: Quartile 3; **^a^** Change at 4 weeks relative to baseline measurements determined using median regression models with bootstrapped estimates (1000 replicates) computed for the 25^th^, 50^th^ and 75^th^ quantiles; **heiQ**: Health Education Impact Questionnaire, missing individual options were replaced with the average score of the specific dimension; **HADS**: Hospital Anxiety and Depression Scale; **NEADL**: Nottingham Extended Activities of Daily Living Scale, missing individual options were replaced with the value 3 (‘On your own easily’); **EQ-5D:** EuroQol health-related quality of life five dimensions questionnaire – number reporting some/moderate problems on each domain. **^:**1-2 cases missing.
